# The *Entamoeba histolytica *genome: primary structure and expression of proteolytic enzymes

**DOI:** 10.1186/1471-2164-8-170

**Published:** 2007-06-14

**Authors:** Manuela Tillack, Laura Biller, Henriette Irmer, Michelle Freitas, Maria A Gomes, Egbert Tannich, Iris Bruchhaus

**Affiliations:** 1 Bernhard Nocht Institute for Tropical Medicine, Bernhard Nocht Str. 74, 20359 Hamburg, Germany; 2 University of Minas Gerais, Dept. Parasitologia, ICB-UFMG, Laboratorio Amebiase, Av. Antonio Carlos, 6627, 31270-901 Belo Horizonte, Brasil

## Abstract

**Background:**

A number of studies have shown that peptidases and in particular cysteine peptidases constitute major pathogenicity factors in *Entamoeba histolytica*. Recent studies have suggested that a considerable number of genes coding for proteolytic enzymes are present within the *E. histolytica *genome and questions remain about the mode of expression of the various molecules.

**Results:**

By homology search within the recently published amoeba genome, we identified a total of 86 *E. histolytica *genes coding for putative peptidases, including 46 recently described peptidase genes. In total these comprise (i) 50 cysteine peptidases of different families but most of which belong to the C1 papain superfamily, (ii) 22 different metallo peptidases from at least 11 different families, (iii) 10 serine peptidases belonging to 3 different families, and (iv) 4 aspartic peptidases of only one family. Using an oligonucleotide microarray, peptidase gene expression patterns of 7 different *E. histolytica *isolates as well as of heat stressed cells were analysed. A total of 21 out of 79 amoeba peptidase genes analysed were found to be significantly expressed under standard axenic culture conditions whereas the remaining are not expressed or at very low levels only. In heat-stressed cells the expression of 2 and 3 peptidase genes, respectively, were either decreased or increased. Only minor differences were observed between the various isolates investigated, despite the fact that these isolates were originated from asymptomatic individuals or from patients with various forms of amoebic diseases.

**Conclusion:**

*Entamoeba histolytica *possesses a large number of genes coding for proteolytic enzymes. Under standard culture conditions or upon heat-stress only a relatively small number of these genes is significantly expressed and only very few variations become apparent between various clinical *E. histolytica *isolates, calling into question the importance of these enzymes in *E. histolytica *pathogenicity. Further studies are required to define the precise role of most of the proteolytic enzyme for amoeba cell biology but in particular for *E. histolytica *virulence.

## Background

The faecal-oral spread protozoan parasite *Entamoeba histolytica *is an important human pathogen. Normally, this parasite resides and multiplies in the large bowel and can persist there for months and years causing only an asymptomatic luminal gut infection. However, occasionally *E. histolytica *penetrates the intestinal mucosa, which leads to ulcerative colitis or it disseminates to other organs, most commonly to the liver, where it induces abscess formation. Cysteine peptidases are considered to play a major role for the pathogenicity of *E. histolytica *as suggested by a large number of *in vitro *and *in vivo *studies [1-9]. Most convincing are results from infections of laboratory animals indicating that *E. histolytica *trophozoites that have reduced cysteine peptidase activity are greatly impaired in their ability to induce amoebic liver abscesses [8,9]. In addition, overexpression of cysteine peptidases led to an increase in cytopathic activity, measured by *in vitro *monolayer disruption, as well as to a significant increase in amoebic liver abscess formation in laboratory animals in comparison to matching controls [10]. Furthermore, the discovery that amoeba cysteine peptidases possess interleukin-1β converting enzyme activity suggests a novel mechanism of these enzymes in amoebic virulence [11].

Homology searches based on the conservation of active site regions revealed that the *E. histolytica *genome contains a multitude of at least 50 genes coding for cysteine peptidases (reviewed by Clark et al [12]). Of these, the majority is structurally related to the C1 papain superfamily, whereas a few others are more similar to family C2 (calpain-like cysteine proteinases), C19 (ubiquitinyl hydrolase), C48 (Ulp1 peptidase), C54 (autophagin), and C65 (otubain), respectively [12].

Phylogenetic analyses of the 37 C1-family members revealed that they represent 3 distinct clades (A, B, C), each consisting of 13, 11 and 13 members, respectively [12]. EhCP-A and EhCP-B family members are organised as classical pre-pro enzymes with an overall cathepsin L-like structure. They differ in length of the pro regions as well as of the catalytic domains and have specific sequence motifs within the N-terminal regions of the mature enzymes. In addition, most members of the EhCP-B contain hydrophobic stretches near or at the C-terminus [12,13]. The primary structure prediction of the 13 EhCP-C members indicated a hydrophobic region located 11 to 28 amino acid residues apart from the N-terminus, which is predicted to form a signal anchor. As there is no example of a structural related cysteine peptidase corresponding to the EhCP-C subfamily, any function of this group of molecules remains to be determined.

In addition, two genes encoding for putative cysteine peptidases of the family C2 (calpain-like proteases) were identified within the genome (EhCALP1 and EhCALP2). These molecules are involved in several cellular processes including signal transduction pathways, remodelling of the cytoskeleton and membranes and apoptosis [14].

Another 4 genes were identified coding for enzymes with homology to the peptidase family C54 also termed autophagins (EhAUTO1-4). The process of autophagy has initially been described in other eukaryotic cells as a rescue mechanism that is induced upon starvation or oxidative stress. It is a process by which cells digest parts of their own cytosolic material. This allows the recycling of molecules under conditions of nutritional limitation and remodelling of intracellular structure for cell differentiation [15-17].

Four other genes putatively encoding cysteine peptidases in *E. histolytica *show homology to members of the C19 and C65 families. These two groups of enzymes are known to be involved in ubiquitin degradation. In addition 3 genes with homology to Ulp1 peptidase (C48 family) were found. Ulp1 is a member of a family of peptidases that control the function of SUMO a small ubiquitin like modifier protein [18].

Only preliminary data are available for other peptidase family members in *E. histolytica*. So far, a collagenase [19], a high molecular weight proteinase [20], a serine-metallo proteinase [21], a tripeptidyl peptidase I [22] and a serine protease [23] have been reported.

In this study we have analysed the genome of *E. histolytica *for the presence of additional peptidases belonging to the aspartate, serine and metallo peptidase families. Furthermore, the expression profile of the amoeba genes for the various proteolytic enzymes was assessed in 7 different *E. histolytica *isolates as well as under heat stress conditions using an oligonucleotide-based microarray and quantitative real time PCR.

## Results

### Peptidase genes in *E. histolytica*

**Figure 1:**
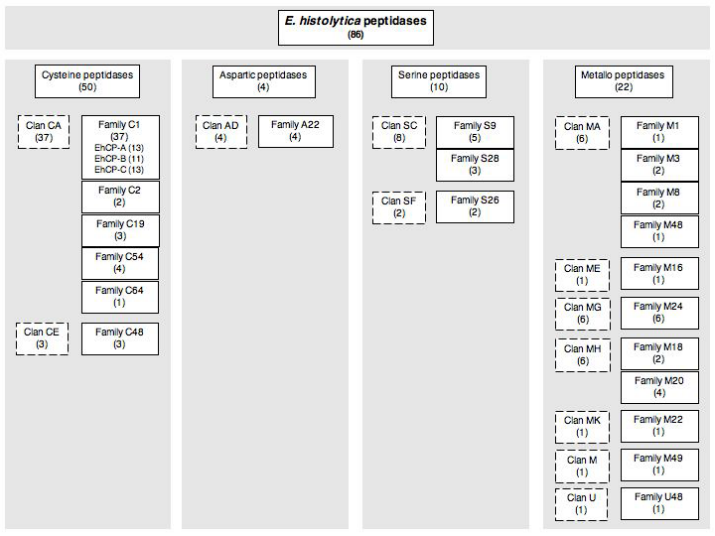
Summary of the peptidase gene families identified within the *Entamoeba histolytica *genome. The identified enzymes were grouped into the corresponding peptidase clans and families according to the MEROPS nomenclature.

Homology search within the *E. histolytica *genome revealed a total of 86 genes coding for putative peptidases. These comprise 50 cysteine peptidases of various families, all of which belonging to clan CA. In addition, 4 aspartic, 10 serine and 22 metallo peptidase genes were identified (Figure 1, Table 1). Structural details of the various *E. histolytica *cysteine peptidases have been described recently [12].

**Table 1:** Peptidases of *Entamoeba histolytica*

	**Protein name**	**Clan, family, subfamily**	**Accession No.**	**Protein length**	**Active site residues**	**Remarks**	**Name, NCBI**
**Cysteine peptidases**							

1	EhCP-A1	CA, C1, A	XP_650156 XM_645064	315	Q_112_C_118_H_259_N_279_	SP: 13, Pro: 80, TM: -	cysteine protease 1
2	EhCP-A2	CA, C1, A	XP_650642 XM_645550	315	Q_112_C_118_H_259_N_279_	SP: 13, Pro; 80, TM: -	cysteine proteinase 2
3	EhCP-A3	CA, C1, A	XP_653254 XM_648162	308	Q_111_C_115_H_251_N_271_	SP: 13, Pro: 79, TM: -	cysteine proteinase acp1
4	EhCP-A4	CA, C1, A	XP_656602 XM_651510	311	Q_112_C_118_H_253_N_273_	SP: 20, Pro: 73, TM: -	cysteine proteinase
5	EhCP-A5	CA, C1, A	XP_650937 XM_645845	318	Q_113_C_119_H_261_N_271_	SP: 20, Pro: 72, TM: -	cysteine proteinase
6	EhCP-A6	CA, C1, A	XP_657364 XM_652272	320	Q_115_C_122_H_261_N_281_	SP: 17, Pro: 79, TM: -	cysteine proteinase
7	EhCP-A7	CA, C1, A	XP_648996 XM_643904	315	Q_112_C_118_H_259_N_279_	SP: 13, Pro: 80, TM: -	cysteine protease 8
8	EhCP-A8	CA, C1, A	XP_657446 XM_652354	317	Q_116_C_122_H_260_N_280_	SP: 15, Pro: 82, TM: -	cysteine protease 9
9	EhCP-A9	CA, C1, A	XP_655675 XM_650583	297	Q_126_C_132_H_269_N_290_	SP: 17, Pro: 90, TM: -	cysteine protease 10
10	EhCP-A10	CA, C1, A	XP_651147 XM_646598	420	Q_185_C_191_H_336_N_357_	SP: 18, Pro: 148, TM: -	cysteine protease 17
11	EhCP-A11	CA, C1, A	XP_651690 XM_646598	324	Q_118_C_124_H_-_N_287_	SP: 17, Pro: 79, TM: -	cysteine protease 19
12	EhCP-A12	CA, C1, A	XP_653823 XM_648731	317	not identified, N_281_	SP: 14, Pro: 83, TM: -	cysteine proteinase
13	EhCP-A13	CA, C1, A	not annotated	250 (IS)	Q_125_C_130_	SP: 18, Pro: 108	
14	EhCP-B1	CA, C1, A	XP_651581 XM_646489	426	Q_105_C_101_H_308_N_328_	SP: 15, Pro: 106, hydroph. C-term.	cysteine proteinase 7
15	EhCP-B2	CA, C1, A	AAO03568	431	Q_145_C_151_H_308_S_328_	SP: 15, Pro: 106, GPI	cysteine protease 11
16	EhCP-B3	CA, C1, A	XP_656747 XM_651655	474	Q_155_C_161_H_304_N_324_	SP: 16, Pro: 107, TM: 444–466	cysteine protease 12-related
17	EhCP-B4	CA, C1, A	XP_648501 XM_643409	379	Q_153_C_159_H_302_N_322_	SP: 16, Pro: 105, TM: 355–377 or GPI	cysteine protease 13
18	EhCP-B5	CA, C1, A	XP_652671 XM_647579	434	Q_151_C_157_H_311_N_326_	SP: 12, Pro: 108, GPI	cysteine protease 14-related
19	EhCP-B6	CA, C1, A	XP_652465 XM_647373	300	Q_84_C_90_H_232_N_252_	SP: 14, Pro: 55, hydroph. C-term.	cysteine protease 15
20	EhCP-B7	CA, C1, A	XP_650400 XM_645308	650	Q-C_171_H_312_N_332_	SP: 18, Pro: 144, hydroph. C-term.	cysteine protease 16
21	EhCP-B8	CA, C1, A	XP_651049 XM_645957	473	Q_110_C_156_H_329_N_249_	SP: 15, Pro: 105, GPI	cysteine protease 18
22	EhCP-B9	CA, C1, A	XP_652993 XM_647901	446	Q_161_C_167_H_328_N_348_	SP: 19, Pro: 112, hydroph. C-term	cysteine protease
23	EhCP-B10	CA, C1, A	XP_648306 XM_643214	372 (IS)	Q_67_C_76_H_244_N_264_	hydroph. C-term.	cysteine protease
24	EhCP-B11	CA, C1, A	XP_648013 XM_642921	133 (IS)			cysteine protease 11-related
25	EhCP-C1	CA, C1, A	XP_654453 XM_649361	586	Q_70_C_76_H-N_345_	SA: 12–34	hypothetical protein
26	EhCP-C2	CA, C1, A	XP_656632 XM_651540	567	Q_87_C_93_H_306_N_326_	SA: 27–49	hypothetical protein
27	EhCP-C3	CA, C1, A	XP_655128 XM_650036	572	Q_94_C_100_H_322_N_337_	SA: 17–39	hypothetical protein
28	EhCP-C4	CA, C1, A	XP_655800 XM_650708	502	Q_32_C_38_H_246_N_271_	SP: 15	hypothetical protein
29	EhCP-C5	CA, C1, A	XP_654800 XM_649708	557	Q_90_C_96_H_302_N_327_	SA: 20–42	hypothetical protein
30	EhCP-C6	CA, C1, A	XP_651553 XM_646461	557	Q_93_C_99_H_293_N-	SA: 14–36	hypothetical protein
31	EhCP-C7	CA, C1, A	XP_657273 XM_652181	595	Q_89_C_95_H_297_N_322_	SA: 19–41	hypothetical protein
32	EhCP-C8	CA, C1, A	XP_655479 XM_652181	627	Q_91_C_96_H_317_N_366_	SA: 29–51	hypothetical protein
33	EhCP-C9	CA, C1, A	XP_655011 XM_649919	518	not identified	SA: 12–34	hypothetical protein
34	EhCP-C10	CA, C1, A	XP_654829 XM_649737	530	Q_87_C_93_H_299_N_324_	SA: 15–37	hypothetical protein
35	EhCP-C11	CA, C1, A	XP_648083 XM_642991	526	not identified	SA: 20–42	hypothetical protein
36	EhCP-C12	CA, C1, A	XP_650829 XM_645737	473	not identified	SA: 26–48, TM: 449–471	hypothetical protein
37	EhCP-C13	CA, C1, A	XP_656556 XM_651464	564	Q_89_C_95_H_266_N_290_	SA: 21–43	hypothetical protein
38	EhCALP1	CA, C2	XP_649922 XM_644830	591	not identified	SP: -, TM: -	calpain-like cysteine protease
39	EhCALP2	CA, C2	XP_657312 XM_652220	473	Q_51_C_57_H_206_N_227_	SP: -, TM: -	calpain family cysteine protease
40	EhUBHY	CA, C19	XP_657356 XM_652264	444	not identified	SP: -, TM: -	peptidase
41	EhUBP	CA, C19	XP_654028 XM_648936	352	N_30_C_35_H_330_D_348_	SP: -, TM: -	ubiquitin-specific protease
42	EhUCH	CA, C19	XP_655880 XM_650788	386	N_37_C_42_H_345_D_360_	SP: -, TM: -	ubiquitin carboxyl-terminal hydrolase
43	EhUlp1-1	CA, C48	XP_650529 XM_645437	197	H_95_D_112_Q_146_C_152_	SP: -, TM: -	Ulp1 protease
44	EhUlp1-2	CA, C48	XP_651052 XM_645960	538	H_399_D_435_Q_482_C_488_	SP: -, TM: -	Ulp1 protease
45	EhUlp1-3	CA, C48	XP_657158 XM_652066	285	H_174_D_191_Q_234_C_240_	SP: -, TM: -	Ulp1 protease
46	EhAUTO1	CA, C54	XP_651386 XM_646294	325	Y_71_C_100_D_251_H_253_	SP: -, TM: -	peptidase
47	EhAUTO2	CA, C54	XP_653798 XM_648706	364	Y_93_C_103_D_278_H_280_	SP: -, TM: -	peptidase
48	EhAUTO3	CA, C54	XP_652043 XM_646951	364	Y_92_C_103_D_279_H_281_	SP: -, TM: -	hypothetical protein
49	EhAUTO4	CA, C54	XP_656724 XM_651632	348	Y_92_C_112_D_265_H_267_	SP: -, TM: -	hypothetical protein
50	EhOTU	CA, C65	XP_654013 XM_648921	259	D_105_C_108_H_212_	SP: -, TM: -	OTU-like cysteine protease

**Aspartic peptidase**							

51	EhAsP22-1	AD, A22, A	XP_654079 XM_648987	340	D_178_D_223_	SP:24 or TM: 7–26 + 7 × TM	Intramembrane protease
52	EhAsP22-2	AD, A22, A	XP_652820 XM_647728	316	D_157_, D_209_	SP: 19 or TM: 2–19 + 8 × TM	Signal peptide peptidase
53	EhAsP22-3	AD, A22, A	XP_657563 XM_652471	320	D_157_D_205_	SA:7–29 + 8 xTM	Signal peptide peptidase
54	EhAsP22-4	AD, A22, A	XP_653696 XM_648604	396	D_245_D_318_	SP: -, 7 × TM	Presenilin 1-related peptidase

**Serine peptidase**							

55	EhSP9-1	SC, S9, C	XP_655265 XM_650173	653	S_514_D_595_H_627_	SP: 15, TM: -	dipeptidyl-peptidase
56	EhSP9-2	SC, S9, C	XP_655222 XM_650130	665	S_526_D_607_H_639_	SP: 15, TM: -	dipeptidyl-peptidase
57	EhSP9-3	SC, S9, C	XP_656380 XM_651288	656	S_516_D_599_H_632_	SP: -, TM: -	prolyl oligopeptidase
58	EhSP9-4	SC, S9, C	XP_649111 XM_644019 XP_648413 XM_643321 XP_655473 XM_650381	669	not identified	SP: 16, TM: -	dipeptidyl-peptidase
59	EhSP9-5	SC, S9	XP_655676 XM_650584	102 (IS)		SP: -, TM: -	prolyl oligopeptidase family
60	EhSP26-1	SF, S26, B	XP_653142 XM_648050	189	S_65_H_106_	SP: -, TM: 34–56, 163–185	signal peptidase (signalase)
61	EhSP26-2	SF, S26	XP_651791 XM_646699	121	not identified	SP: -, TM: -	microsomal signal peptidase
62	EhSP28-1	SC, S28	XP_656762 XM_651670	457	S_165_D_395_H_421_	SP: 15, TM: -	serine peptidase
63	EhSP28-2	SC, S28	XP_648991 XM_643899	480	S_165_D_418_H_444_	SP: 15, TM: -	serine peptidase
64	EhSP28-3	SC, S28	XP_652089 XM_646997	466	S_155_D_404_H_431_	SP: -, TM: -	serine peptidase

**Metallo peptidases**							

65	EhMP1-1	MA, M1	XP_652558 XM_647466	827	H_295_E_296_H_299_E_318_Y_481_	SP: -, TM: -	aminopeptidase
66	EhMP3-1	MA, M3	XP_649877 XM_644785	675	H_463_E_464_H_467_E_493_	SP: -, TM: -	oligopeptidase A
67	EhMP3-2	MA, M3	XP_649600 XM_644508	710	H_498_E4_99_H_502_E_527_	SP: -, TM: -	oligopeptidase A
68	EhMP8-1	MA, M8	XP_655394 XM_650302	643	H_206_E_207_H_210_H_270_M_281_	SP: 18, TM: 605–627	leishmaniolysin-related peptidase
69	EhMP8-2	MA, M8	XP_652632 XM_647540	662	H_207_E_208_H_211_H_267_M_278_	SP: -, TM: 598–620	leishmaniolysin-related peptidase
70	EhMP48-1	MA, M48, A	XP_648770 XM_643678	416	H_274_E_275_H_278_E_353_	SA: 4–21 + 6 × TM	CAAX prenyl protease
71	EhMP16-1	ME, M16, C	XP_654849 XM_649757	970	H_59_E_62_H_63_E_137_E_158_	SP: -, TM: -	Zn-dependent peptidase, eupitrilysin
72	EhMP24-1	MG, M24, A	XP_651539 XM_646447	409	H_165_D_186_D_197_H_266_E_299_E_394_	SP: -, TM: -	methionine aminopeptidase
73	EhMP24-2	MG, M24, B	XP_657085 XM_651993	471	H_244_D_265_D_276_H_354_H_358_H_365_E_401_E_441_	SP: -, TM: -	Xaa-Pro dipeptidase
74	EhMP24-3	MG, M24, B	XP_654211 XM_649119	563	H_364_D_384_D_396_H_461_H_465_H_470_E_491_E_505_	SP: -, TM: -	aminopeptidase
75	EhMP24-4	MG, M24, B	XP_650646 XM_645554	559	H_346_D_366_D_378_H_443_H_447_H_452_E_473_E_487_	SP: -, TM: -	aminopeptidase
76	EhMP24-5	MG, M24, B	XP_649980 XM_644888	589	H_369_D_389_D_401_H_466_H_475_E_496_E_510_	SP: -, TM: -	aminopeptidase
77	EhMP24-6	MG, M24, B	XP_653331 XM_648239 XM_649891 XM_644799	371	not identified	SP: -, TM: -	peptidase
78	EhMP18-1	MH, M18	XP_656618 XM_651526	435	H_79_D_81_D_222_E_258_E_259_D_307_H_402_	SP: -, TM: -	aminopeptidase
79	EhMP18-2	MH, M18	XP_650466 XM_645374	431	H_81_D_82_D_228_E_262_E_263_D_311_H_401_	SP: -, TM: -	aspartyl aminopeptidase
80	EhMP20-1	MH, M20, B	XP_656428 XM_651336	379	H_75_D_133_E_163_E_164_D_186_H_353_	SP: -, TM: -	peptidase T
81	EhMP20-2	MH, M20, B	XP_650152 XM_645060	401	H_79_D_139_E_172_E_173_D_195_H_376_	SP: -, TM: -	peptidase T
82	EhMP20-3	MH, M20, C	XP_656545 XM_651453 XP_655596 XM_650504 XP_652163 XM_647071	516	H_95_D_97_D_134_E_165_E_166_D_192_H_490_	SP: -, TM: -	aminoacyl-histidine dipeptidase
83	EhMP20-4	MH, M20, C	XP_655616 XM_650524	505	H_95_D_97_D_-_E_154_E_155_D_181_H_477_	SP: -, TM: -	aminoacyl-histidine dipeptidase
84	EhMP22-1	MK, M22	XP_652292 XM_647200	335	unknown	SP: -, TM: -	glycoprotein endopeptidase
85	EhMP49-1	M, M49	XP_654273 XM_649181	645	H_412_E_413_H_417_E_467_	SP: -, TM: -	dipeptidyl-peptidase III
86	EhU48-1	U, U48	XP_656466 XM_651374	216	H_274_E_275_H_278_E_353_	SA: 4–26 + 6 × TM	CAAX prenyl protease

Primary structure prediction of the other 3 groups of proteolytic enzymes are as follows:

### Aspartic peptidases

The 4 aspartic peptidases (EhAsP22-1 to EhAsP22-4) share 30 to 40% sequence identity and are homologous to intramembrane-cleaving peptidases (clan AD, family A22). All of them have the specific active site residues TyrAsp and GlyLeuGlyAsp and contain 7 or 8 transmembrane domains but only EhAsP22-1 and EhAsP22-2 have recognizable signal peptides, whereas EhAsP22-3 contains a predicted signal anchor motif. EhAsp22-1, -2 and -3 have significant homology to signal peptide peptidases of various organisms including *Trypanosoma cruzi *and *Arabidopsis thaliana *and in addition, EhAsP22-1 and EhAsP22-3 contain the signal peptide peptidase-specific motif GlnProAlaLeuLeuTyr [24,25]. The primary structure of EhAsp22-4 revealed highest identity (35–40%) to putative presenilins of various organisms including *Dictyostelium discoideum*, *Arabidopsis thaliana *and *Homo sapiens*, but a signal peptide or signal anchor is absent.

### Serine peptidases

Of the 10 *E. histolytica *genes coding for putative serine peptidases, 5 are predicted to belong to clan SC, family S9 (Figure 1, Table 1), with the active site residues Ser, Asp, His. According to the amino acid residues adjacent to the active site Ser (GG**S**YGG), EhSP9-1, -2, and -3 can be grouped into subfamily C. The sequences of EhSP9-1 and EhSP9-2 are identical except for a 12 amino acid insertion present in EhSP9-2. In contrast, EhSP9-3, -4 or -5 share only 20% sequence identity with EhSP9-1 or EhSP9-2. The active site residues of EhSP9-4 are not conserved and for EhSP9-5 only a partial sequence of 102 amino acid residues is available. Thus, a reliable assignment of these two enzymes to a specific S9 subfamily is not possible. Signal peptides were identified only for EhSP9-1, EhSP9-3 and EhSP9-4, respectively.

Another 3 enzymes were classified into clan SC but represent most likely members of family S28 of serine peptidases (EhSP28-1, -2, -3), which are also known as lysosomal Pro-Xaa carboxypeptidases. All 3 molecules have a predicted signal peptide and are of similar size comprising betwen 457 and 480 amino acid residues. EhSP28-1 (EhSp1) and EhSP28-2 (EhSp2) have been previously characterized [23]. Both are highly similar as they share 89% sequence identity, but only 35% to EhSP28-3.

Two other serine peptidases (EhSP26-1 and EhSP26-2) have homology to members of the signal peptidase family S26B (clan SF) containing the catalytic dyad Ser and His. EhSP26-1 has a calculated molecular mass of approximately 20 kDa and contains 2 hydrophobic regions located near the N- and C-terminus, respectively. Sequence similarity to other members of this family is approximately 45%. In contrast, EhSP26-2 shares only 20% sequence identity with members of the S26 family. Moreover, it does not contain predicted transmembrane regions and the active site is not conserved.

### Metallo peptidases

A considerable number of 22 *E. histolytica *genes are predicted to encode putative metallo peptidases. These are relatively diverse and can be attributed to 7 different clans and 11 different families (Figure 1, Table 1). Six of the enzymes group into clan MA, with the characteristic zinc binding-motif consisting of two histidine residues encompassing the sequence HEXXH. One member is assigned to family M1 (EhMP1-1) and two others to family M3 (EhMP3-1, EhMP3-2). The latter are known as Glu-zincins with the third Zn-binding site being a glutamate residue.

Another two clan MA members (EhMP8-1, EhMP8-2) are homologous to metzincins, which are characterized by a C-terminal His residue being a third zinc-binding site. The two enzymes share 34% sequence identity and both contain a predicted C-terminal transmembrane region but only EhMP8-1 has a signal sequence.

A further clan MA member belongs to family M48 (EhMP48-1) and contains a predicted signal anchor and 6 additional transmembrane domains. The structure of EhMP48-1 is homologous to ste24, an endopeptidase from yeast. Like the yeast enzyme, the amoeba molecule contains the conserved HEXXH zinc-binding motif, located between the fourth and the fifth transmembrane domain.

Another putative metallo peptidase was assigned to clan ME, family M16C containing the characteristic zinc-binding motif HXXEH. Members of this family are falcilysin from *Plasmodium falciparum*, eupitrilysin from *Homo sapiens *and CYM1 peptidase from *Saccharomyces cerevisiae*.

A group of 6 enzymes (EhMP24-1 to EhMP24-6) was predicted to constitute metallo peptidases of clan MG, family 24, which usually represent cytosolic exopeptidases that require co-catalytic ions such as cobalt or manganese. Another 6 peptidases were identified, with homology to metallo peptidases of clan MH. Of these, 2 constitute most likely aspartyl aminopeptidases belonging to family M18 (EhMP18-1, EhMP18-2). They share 40% identity and approximately 35% with members of this family from other organisms. The other 4 amoeba enzymes of clan MH were attributed to family 20 (EhMP20-1 to EhMP20-4). In general, enzymes of this family hydrolyse the late products of protein degradation to complete the conversion of proteins into free amino acids.

The deduced amino acid sequence of a further amoeba gene revealed homology to clan MK, family 22 of metallo peptidases. The only enzyme belonging to this family known so far is the *O*-sialoglycoprotein endopeptidase from *Pasteurella haemolytica*. At present, the nature of the active site residues is unknown [26]. Like the amoeba homologue, the bacterial peptidase does not possess a signal peptide.

In addition, one *E. histolytica *enzyme was identified belonging to family M49, clan M. The mammalian homologues are cytosolic dipeptidyl peptidases, which sequentially release N-terminal dipeptides [27]. Moreover, an enzyme designated EhU48-1 were annotated, which is similar to EhMP48-1. Like EhMP48-1, it contains a signal anchor sequence and six transmembrane domains. Nevertheless, homology search grouped this peptidase into the U48 family. However, the specificities of the two families are overlapping but not identical [28,29].

### Peptidase gene expression of various *E. histolytica *isolate under standard axenic culture conditions

**Figure 2:**
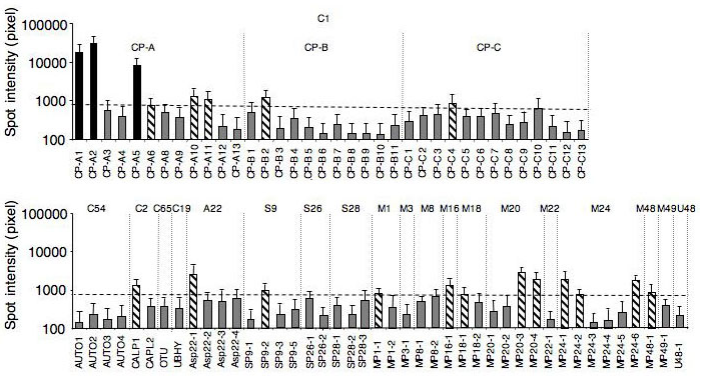
Expression of *E. histolytica *peptidase genes of the *E. histolytica *isolate HM-1:IMSS as determined by microarrays. Error bars represent the standard error of the mean of nine hybridizations (biological replicates).

To allow detailed expression analyses of the various *E. histolytica *peptidase genes, a small microarray was designed. This array contains 86 specific oligonucleotides representing 4 different *E. histolytica *houskeeping genes, 3 peptidase-inhibitor genes as well as 79 of the 86 identified peptidase genes. Genes coding for the serine peptidase EhSP9-4 or for the cysteine peptidases EhCP-A7, EhUBP, EhUCH, EhUlp-1, EhUlp-2 and EhUlp-3, respectively, were not included because it was either not possible to design a specific oligonucleotide or they were identified after the array was already spotted. In a first attempt, labelled cDNA from the widely used laboratory strain HM-1:IMSS was hybridized to the array (Figure 2). The results from multiple experiments using RNA preparations from cells grown under standard axenic culture conditions were highly reproducible and indicated that only 3 peptidase genes were expressed at high levels (mean spot intensity >8000), all of them encoding cysteine proteinases (EhCP-A1, EhCP-A2, EhCP-A5). A set of 17 peptidase genes revealed intermediate expression levels (mean spot intensity 800 to 3000). This group comprised the genes for the cysteine peptidases EhCP-A6, -A10, -A11, -B2, -C4 and EhCALP1, the aspartic peptidase EhAsP22-1, the serine peptidase EhSP9-2 and the metallo peptidases EhMP1-1, 16-1, 18-1, 20-3, 20-4, 24-1, 24-2, 24-6 and 48-1, respectively. All other peptidase genes were expressed at levels below the detection limit of Northern blots (mean spot intensity <700). The reliability of the results obtained by array hybridization was confirmed by qRT-PCR using a set of 22 pairs of primers amplyfing cDNAs of the 3 highly expressed genes as well as a representative number of the intermediate or low expressed peptidase genes (Table 2).

**Table 2:** Confirmation of microarray results via real time PCR.

**Gene accession**	**Gene name**	**Microarray data**		**Real time PCR data**
			
		Signal intensity (pixel)	relative expression	relative expression
**High expression**				

	*actin*	33512	1	1
XM_645064	*ehcp-a1*	17691	0.528	0.853
XM_645550	*ehcp-a2*	32474	0.969	0.853
XM_645845	*ehcp-a5*	8628	0.257	0.368

**Intermediate**				

XM_648987	*ehasp22-1*	2156	0.064	0.020
XM_652272	*ehcp-a6*	802	0.024	0.026
XM_650130	*ehsp 9-2*	974	0.029	0.035
XM_651453 XM_650504 XM_647071	*ehmp20-3*	2838	0.085	0.360
XM_651374	*ehmp48-1*	895	0.027	0.020

**Low**				

XM_648162	*ehcp-a3*	549	0.016	0.000
XM_651510	*ehcp-a4*	414	0.012	0.013
XM_652354	*ehcp-a8*	510	0.015	0.010
XM_647728	*ehasp 22-2*	458	0.014	0.000
XM_652471	*ehasp 22-3*	450	0.013	0.010
XM_648604	*ehasp 22-4*	516	0.015	0.000
XM_651670	*ehsp28-1*	380	0.011	0.010
XM_646997	*ehsp 28-4*	525	0.016	0.020
XM_650302	*ehmp8-1*	542	0.016	0.015
XM_647540	*ehmp8-2*	686	0.020	0.010
XM_649181	*ehmp49-1*	406	0.012	0.023
XM_647901	*ehcp-b9*	144	0.004	0.000
XM_651336	*ehmp20-1*	282	0.008	0.000
XM_643899	*ehsp 28-2*	230	0.007	0.009

Gene expression profile of selected peptidases in *E. histolytica *isolate HM-1:IMSS.

For microarray analysis nine independent experiments were performed. The signal intensity is indicated in pixel. For real time PCR analysis all primer sets were run in duplicates. Two biological replicates were investigated. The expression of actin as normalizer was set to 1. The relative expression of the peptidases were related to actin.

In order to determine the extend of inter-strain variation in the expression of peptidase genes, HM-1:IMSS was compared with 6 different *E. histolytica *isolates all of them cultivated under axenic conditions. These isolates originated from different parts of the world and were obtained from patients with different forms of amoebic disease or in at least one case from an asymptomatic *E. histolytica *carrier. Pairwise comparison of the various isolates with HM-1:IMSS revealed only minor differences in the expression of the various peptidase genes (Table 3). Three isolates including the one from an asymptomatic carrier showed no differences and two isolates differed only in the expression of one gene. In isolate HK-9, expression of the of the gene for cysteine peptidase EhCP-A5 was decreased by 2.3 fold and in isolate DRP expression of the the gene for the metallo peptidase EhM48-1 was increased by 5.2 fold. The only exception was isolate EGG, which revealed differences in expression for 4 peptidase genes. This isolate was obtained from a patient who simultaneously developed amoebic colitis and liver abscess. Compared to HM-1:IMSS isolate EEG showed decreased expression of the genes for the cysteine peptidase EhCP-A1 as well as for the metallo peptidase EhMP20-3 by about 2 fold, and an increase in the expression of the genes for serine peptidase EhSP9-2 and for the metallo peptidase EhMP20-1 by about 2.8 and 8.6 fold, respectively.

**Table 3:** Genes differentially expressed in various *E. histolytica *isolates in comparison to the *E. histolytica *isolate HM-1:IMSS.

**Gene accession**	**Gene name**	**Change of signal intensity HM-1:IMSS/strain of interest (pixel)**	**Fold change**	***p*-value**
*Expression of HM-1:IMSS/NIH:200*				
No				

*Expression of HM-1:IMSS/HK-9*				

XM_645845	*EhCP-A5*	5.860 → 2.500	2.3 × decrease	0.0001

*Expression of HM-1:IMSS/EGG*				

XM_651336	*EhMP20-1*	570 → 4.900	8.6 × increase	0.0004
XM_650130	*EhSP9-2*	1.700 → 4.700	2.8 × increase	< 0.0001
XM_645064	*EhCP-A1*	7.300 → 3.700	2.0 × decrease	0.0006
XM_651453	*EhMP20-3*	1.600 → 700	2.3 × decrease	0.0001

*Expression of HM-1:IMSS/DRP*				

XM_643678	*EhM48-1*	850 → 4.400	5.2 × increase	< 0.0001

*Expression of HM-1:IMSS/452*				

*No*				

*Expression of HM-1:IMSS/32*				

*No*				

The gene accession number, gene name, fold change and *p*-value are shown. All transcripts are statistically significant, having a *p*-value < 0.001.

### Peptidase gene expression in response to heat stress

Previous studies have suggested that the level of expression of a number of cysteine peptidase genes is sensitive to heat shock [30,31]. To further characterize the influence of heat stress on the expression pattern of the various *E. histolytica *peptidase genes, amoeba were cultured at 42°C for 4 hours and compared with amoebae cultivated under standard culture conditions at 36°C. The results indicated that only 5 of the 79 peptidase genes investigated were differentially expressed upon heat shock. The amount of RNA for the highly expressed genes *ehcp-a1 *and *ehcp-a2 *was found to be decreased by about 6 and 4 fold, respectively, whereas the expression of *ehcp-a5*, *ehcp-a6 *or *ehmp8-2 *was increased by approximately 2 fold (Table 4). Similar results were obtained by qRT-PCR. However, there were no significant differences in expression for the remaining 74 peptidase genes.

**Table 4:** Genes differentially expressed in *E. histolytica *HM-1:IMSS isolate under heat shock.

**Gene accession**	**Gene name**	**Change of signal intensity 36°C → 42°C (pixel)**	**Fold change**	***p*-value**
*Expression of HM-1:IMSS 36°C/HM-1:IMSS 42°C*				

XM_645845	*EhCP-A5*	14.000 → 32.000	2.3 × increase	0.0001
XM_652272	*EhCP-A6*	1.050 → 2.200	2.1 × increase	0.0001
XM_647540	*EhMP8-2*	680 → 1.500	2.2 × increase	< 0.0001
XM_645064	*EhCP-A1*	25.000 → 4.000	6.3 × decrease	< 0.0001
XM_645550	*EhCP-A2*	45.000 → 11.000	4.1 × decrease	< 0.0001

Only those genes are listed that show a differential expression pattern during heat shock. The gene accession number, gene name, signal intensity, fold change and *p*-value are shown. All transcripts are statistically significant, having a *p*-value < 0.001.

## Discussion

In an attempt to annotate all *E. histolytica *peptidase genes, a total of 86 putative or known proteolytic enzymes were identified within the *E. histolytica *genome. Such a great number of peptidase genes is not unusual for protozoans. So far, 110 annotated peptidases were found for *Plasmodium falciparum *and 70 for *Giardia lamblia*. *Entamoeba*, *Plasmodium *and *Giardia *contain aspartic peptidases of the A22 family. In addition, *P. falciparum *contains genes belonging to the A1 family known as plasmepsins. Of the various cysteine peptidases, the autophagin-like as well as the OTU-like enzymes are only present in *E. histolytica*. On the other hand, several cysteine peptidase families found in *P. falciparum*, such as C2, C12, C13, C14, C44 and C56 have no counterpart in *Entamoeba*. Regarding the serine and metallo peptidases no striking differences between the families of *Entamoeba*, *Plasmodium *and *Giardia *became obvious, except 10 additional peptidase families, that are peculiar for *P. falciparum*. The leishmanolysin-like peptidases of the M8 family are specific for *E. histolytica *and absent in *P. falciparum *and *G. lamblia*, respectively.

Since only a fraction of the 86 putative amoeba peptidases have been biochemically and functionally characterized so far, the function and localization of most of the molecules can only be predicted from the deduced primary structure.

All four aspartic peptidases identified within the *Entamoeba histolytica *genome may belong to intramembrane-cleaving proteases, which usually perform downstream functions such as cell signalling, regulation and intercellular communications [32]. So far, three families of peptidases are known to promote intramembrane cleavage. These are metallo peptidases represented by the human site-2 protease [33], serine peptidases represented by *Drosophila melanogaster *rhomboid-1 [34], and aspartic proteases including human presenilins [35], as well as signal peptide peptidases [24]. Within the *Entamoeba *genome, homologous to only the presenilins and signal peptide peptidases have been found.

One of the amoebic aspartic peptidases (EhAsp22-4) shows highest identity to presenilins. So far, the physiological function of presenilins is not fully understood. Presenilin is one of the subunits that form a multiprotein complex called gamma-secretase [36]. Homologues are found in various organisms of different origin such as *Caenorhabditis elegans*, *Drosophila melanogaster*, and even plants [37,38]. It has been shown that mutations within this protein are associated with Alzheimer's disease [36]. However, homologues to other subunits of this complex, such as nicastrin have not been identified within the *Entamoeba *genome. EhAsp22-4 contains seven putative transmembrane domains. Interestingly, the active site residues were found within one predicted outside loop of the protein. This is different to the other known presenilins [39].

The 11 identified serine peptidases can be grouped into 3 families. The two amoeba serine peptidases characterized so far, belong to family S28, previously designated EhSP1 and EhSP2 and now renamed EhSP28-1 and EhSP28-2 [23]. Biochemical analysis revealed that these peptidases prefer the substrate Suc-AAF-AMC. This enzymatic feature is identical to that of the *E. histolytica *tripeptidyl serine peptidase purified by Flockenhaus and colleagues [22]. Unfortunately, no sequence of the purified tripeptidyl peptidase is available. In addition to these two described serine peptidases, one more serine peptidase gene belonging to family S28 has been identified. Microarray analysis indicated that EhSP28-1, EhSP28-2 and EhSP28-3 are not expressed or expressed at a very low level, which is in contrast to the results of Barrios-Ceballos and colleagues [23]. They postulated that the identified peptidase activity corresponds to EhSP28-2 and that this protein is associated with the trophozoite membrane. Using bioinformatic tools, no hydrophobic stretches or transmembrane domains could be deduced within EhSP28-2. Due to these controversial results, the amoeba serine peptidases require further investigation.

The function of the two S26 family serine peptidases identified within *E. histolytica *is unknown. Peptidases of this family are usually membrane proteins and their function is the processing of newly synthesised secretory proteins. They remove the hydrophobic, N-terminal signal peptides as the proteins are translocated across membranes [40].

Four genes belong to the S9 family (homologous to dipeptidyl-peptidases). One peptidase (SP9-2) shows the highest expression of all serine peptidases analysed in this study. However, the function of these enzymes in *E. histolytica *remains to be determined.

It is likely that some of the *E. histolytica *serine peptidases might play a role during the encystation process is it was shown for *E. invadens*, the in vitro model organism for en- and excystation [41]. Unfortunately, the serine peptidases involved in the *E. invadens *encystations processes have not been identified so far.

A total of 22 genes were identified encoding metallo peptidases, which are predicted to belong to 11 different families. The members of two of the identified metallo peptidase families contain transmembrane domains. These are the leishmanolysin-like peptidases and the CAAX prenyl peptidases. EhMP8-1 and EhMP8-2 are homologous to leishmanolysin found in kinetoplastids. Leishmanolysin occurs mainly as a heavily-glycosylated protein that is attached to the outer membrane of *Leishmania *promastigates by a glycosylphosphatidylinositol anchor. It has been demonstrated that leishmanolysin plays a role in resistance of promastigotes to complement-mediated lysis and in receptor-mediated uptake of the parasite by phagocytic host cells [42]. There are other eukaryotes, including *Caenorhabditis elegans*, *Drosophila melanogaster *and *Homo sapiens *that have homologues of this protein. Nevertheless, highest degree of sequence similarity to the classical leishmanolysin is found for the enzymes of *E. histolytica *and *Dictyostelium discoideum*. However, the proteins of these two organisms have not been characterised so far.

Interestingly, under standard axenic culture conditions only a relatively small number of peptidase genes is significantly expressed. The results are in agreement with a recent study by Ehrenkaufer et al., in which the expression pattern of 38 of the 50 different cysteine peptidase genes were analysed in the standard laboratory *E. histolytica *isolate HM-1:IMSS [43]. However, in contrast to the results presented here, Ehrenkaufer et al., found differences in the expression of a considerable number of peptidase genes when recent clinical isolates were compared with strain HM-1:IMSS. The discrepancy between the two studies is most likely due to differences in the culture media used. In the study presented here, *all E. histolytica *isolates were grown under axenic conditions in a monophasic medium, whereas Ehrenkaufer et al. cultured their recent clinical isolates xenically using a diphasic medium and compared the results with HM-1:IMSS grown under axenic condition in a monophasic medium. As proteolytic enzymes are considered to be involved in nutrition uptake and digestion, differences in the composition of the culture medium and in particular the presence of microorganisms should considerably influence expression of petidase genes in Entamoeba.

However, questions remain about possible functions of all the different peptidases present in *E. histolytica*. At least some of them may be involved in encystation- or exystation processes, as described for *E. invadens *or for a cathepsin C like peptidase of *G. lamblia*, which is involved in processing of cyst-wall specific proteins [44]. Aggressive and invading *Entamoeba *trophozoites should be endowed with adequate mechanisms that ensure their protection against host defence strategies. In this study, the trophozoites were exposed to a temporary heat stress, which partly mimics the situation during tissue invasion. Heat stressed amoebae revealed downregulation of the genes for EhCP-A1 and EhCP-A2 and elevated expression of the genes for EhCP-A5, EhCP-A6 and EhMP8-2, respectively, which is in accordance with a recent report by Weber and colleagues [31]. As EhCP-A6 and EhMP8-2 are expressed at very low levels during *in vitro *cultivation, these enzymes are obviously not essential for parasite growth at least at standard culture conditions. It has been postulated that the upregulation of the gene for EhCP-A6 during heat stress is due to its potential role in the degradation of damaged proteins [31]. Recently, in few other studies, regulation of peptidase expression in response to various conditions has been described. In HM-1:IMSS clone L6, which is deficient in virulence, phagocytosis as well as cysteine peptidase activity, expression of the genes for EhCP-A1, EhCP-A2 and EhCP-A5 was significantly decreased [45]. In contrast, during intestinal colonisation expression of the genes for EhCP-A1, EhCP-A4 and EhCP-A6 was found to be increased [46]. This further highlights the importance of peptidases for *E. histolytica *pathogenicity.

## Conclusion

Under standard culture conditions only a relatively small number of at least 86 identified peptidase genes is expressed and only very few variations become apparent between various clinical *E. histolytica *isolates. Nevertheless, here and in few other studies, it was shown that the peptidase expression can be regulated in response to various conditions. Therefore, further studies are necessary to understand the role of all or at least most of the peptidases in the biochemistry and especially for the virulence of *E. histolytica*.

## Methods

### *E. histolytica *isolates and parasite culture

Seven *E. histolytica *isolates were used in this study. Strain HM-1:IMSS was isolated in 1967 from a patient with amoebic dysentery, strain NIH:200 was isolated in 1949 from a patient with colitis, strain HK-9 was isolated from a patient with amoebic dysentery (year unknown), strain DRP was isolated in 1985 from a patient with an amoeboma, strain EGG was isolated in 1988 from a patient with colitis and amoebic liver abscess, strain 452 was isolated in 1983 from an asymptomatic carrier. Origin of strain 32 is unknown. HM-1:IMSS, NIH:200 and HK-9 are standard laboratory strains obtained from the American Type Culture Collection. They were original isolated in Mexico, India and Korea respectively. All other strains were isolated in Brasil and kindly provided by Prof. E. F. Silva, University of Minas Gerais, Belo Horizonte, Brasil. Different genotypes of the *E. histolytica *strains were confirmed by PCR-based genotyping based on variation in the numbers of short tandem repeats that are linked to *E. histolytica *tRNAs [47]. Trophozoites of the various isolates were cultured axenically in TYI-S-33 medium supplemented with 10% adult bovine serum [48]. Cells were harvested by chilling on ice and subsequent centrifugation at 430 × *g *at 4°C for 5 min. The resulting pellet was washed twice with phosphate-buffered saline (6.7 mM NaHPO_4_, 3.3 mM NaH_2_PO_4_, 140 mM NaCl, pH7.2). For heat shock experiments incubation temperature of cultures was shifted from 36°C to 42°C for 4 hours.

### Identification of peptidase homologous of *E. histolytica*

Conserved domains of cysteine-, serine-, metallo-, or aspartic-peptidases were used for homology search [49] against the *E. histolytica *genome as provided by The Sanger Centre and The Institute of Genomic Research [50-52]. With the help of MEROPS [53] the identified enzymes were grouped into the corresponding peptidase clans and families.

### Microarray design

For microarray experiments a 60-base oligonucleotide array was designed containing probes for 79 of the 86 identified putative peptidase genes. The various oligonucleotides contain similar GC-contents of 35.5% and an average T_m _of 71.6°C, with a standard deviation of 1.17 (range 66–74°C). The oligonucleotides were designed and synthesized by Eurogentec. Each oligonucleotide was printed in quadruplicate on glass slides (Advalytix Epoxy AD100) in a concentration of 50 μM. The spotting procedure was done in cooperation with the University of Marburg (Genomic Solutions OmniGrid). The oligonucleotide sequences are listed [see Additional file 1].

### RNA isolation, microarray hybridization, sample labelling, and visualization

Total amoeba RNA was isolated using TRIZOL reagent (InVitrogen) according to standard protocols. For microarray analysis 5 μg of total RNA was used. Two biological replicates including dye swap experiments were performed. The reverse transcription of RNA into cDNA was performed according to the Atlas Superscript Fluorescent Labeling Kit (TaKaRa) followed by indirect labelling.

The cDNA labelling was performed with the Cy3- and Cy5-monoreactive dyes (Amersham). In a typical oligoarray the control cDNA was labelled with Cy3, while the experimental second cDNA was labelled with Cy5 (and vice versa for dye swap experiment). Prehybridization and hybridization was performed using standard protocols.

### Microarray data analysis

Each array was scanned at 550 nm (Cy3) and at 650 nm (Cy5) at a resolution of 5 μm. Calculation and output of the data was done using ScanArray software, version 3.0 (PerkinElmer). For calculation, the mean signal intensity (pixel) minus local background (pixel) of each spot was used. Flagged spots were eliminated. Two methods for normalizing the data were applied. i) normalization among the totality of genes and ii) normalization among housekeeping genes. For the first normalization method, the signal intensities of each spot of the experiment as well as of each spot of the control were totalised. The sum of the experiment over the sum of the control gives the normalization factor. In order to normalise the control, the signal intensity of each spot of the control was divided by this normalization factor. For housekeeping normalization, the calculation was performed in the same manner as described above but on the basis of the housekeeping gene spot intensities of the control. Spots with a signal intensity (pixel) = 300 were excluded from data analysing. Genes with a ratio of more than 2 and less than 0.5 were considered as differentially expressed.

### Quantitative RT-PCR

In order to validate the results obtained by microarray analyses, quantitative RT-PCR (qRT-PCR) was performed by random sampling. Sense and antisense primers were designed to amplify approximately 100 base pairs [see Additional file 2]. These primers were designed independently from the oligonucleotides used on the microarray. Thus, they represent different regions of the same gene. cDNA synthesis was carried out with SuperScriptIII Reverse Transcriptase (InVitrogen). In a final volume of 20 μl, 1 μg of RNase-free DNase-treated total RNA was mixed with 5 × First-Strand buffer, 500 μM dNTPs, 500 nM OdT-T71 (5'-GAG AGA GGA TCC AAG TAC TAA TAC GAC TCA CTA TAG GGA GAT_24_), 2 mM DTT, 40 U RnaseOut (Invitrogen) and SuperScriptIII (200 U/μl). Incubation was performed for 1 h at 42°C. Quantitative amplification was performed in a Rotor-Gene (Corbett) using RealMasterMix (Eppendorf) SYBR Green kit. 1 μl of the synthesized cDNA was mixed with 2,5× RealMasterMix/20 × SYBR, 5 pmol/μl of the respective sense-primer and antisense-primer to a final volume of 20 μl. Amplification conditions were as follows: 35 cycles at 95°C for 15 s, 58°C for 20 s and 68°C for 20 s and an adjacent melting step (42°C–95°C). Two biological replicates were analysed in triplicate. Relative quantification was carried out with the use of the delta delta ct method provided by the Rotor-Gene software [54] and *E. histolytica *actin gene RNA as normalizer.

## Authors' contributions

IB and ET conceived the study. IB coordinated the study and performed the data analysis together with MT. IB and ET drafted the manuscript. MT, LB, MAG, MF, and HI carried out the laboratory component. MT helped to draft the manuscript. All authors read and approved the final manuscript.

## Supplementary Material

Additional File 1 List of oligonucleotides used for the microarray design. The table shows a list of all oligonucleotides present of the microarray used. Click here for file

Additional File 2 List of oligonucleotides used for RT-PCR. The table shows a list of all oligonucleotides used for RT-PCR. Click here for file
