# Supplementary material for: The Entamoeba histolytica genome: primary structure and expression of proteolytic enzymes
Source: BMC Genomics. 2007 Jun 14;8:170. doi: 10.1186/1471-2164-8-170 (PMC1913524; doi:10.1186/1471-2164-8-170)
Supplement: Additional File 1 — List of oligonucleotides used for the microarray design. The table shows a list of all oligonucleotides present of the microarray used. [file 1471-2164-8-170-S1.doc]

Additional file 1: List of oligonucleotides used for the microarray design

| **No** | **Protein**  **name** | **Spot**  **position** | **Accession No.** | **Oligonucleotide sequence (5’—3’)** |
| --- | --- | --- | --- | --- |
| 1 | AsP22-1 | A2 | XM_648987 | GAAGTTATGTTGACAGTAGCAACTCATGTTGACGGACCAATCAAATTTATCTTCCCTAAA |
| 2 | AsP22-2 | A4 | XM_647728 | ATATTATTGATATGGAATTATCAAATAGGCATACCTGTGTTACTGTGCATAGTTCCAGCA |
| 3 | AsP22-3 | A5 | XM_652471 | ACAAATTCTCTTCCTGAACTTAGTCTACCTTCTGACTTCTCACATGAATCTCCTGTCTTT |
| 4 | AsP22-4 | A3 | XM_648604 | ATGTTGATTCAAACCCATTCACTCTATCATCTACACATGGAAAAGGAGTAAAGTTAGGAC |
| 5 | EhCP-A1 | A6 | XM_645064 | TCACTGTCATTTTGATGTTTTATATTGGATATGGGATTGATTTCAATACATGGGTTGCCA |
| 6 | EhCP-A2 | A7 | XM_645550 | ATGTTTGCTTTTATTTGTTTACTTGCTATTGCAAGTGCTATTGATTTCAATACATGGGCT |
| 7 | EhCP-A3 | A8 | XM_648162 | CAACAACATGTGTGGTATTGGAAGAGATTCTAACTATCCAACCGGAGTCAAGTTAATTTA |
| 8 | EhCP-A4 | A9 | XM_651510 | TTCACTCCAAAAGTTCAAACTACTGGTTTAACTCATGTTACTCCAACTGAAGAAGCTTTA |
| 9 | EhCP-A5 | A10 | XM_645845 | TGCTGTTAAAATTACTGGACAAAAATTAGTTAGACCAGGAAGTGAAAAAGCACTTATGCG |
| 10 | EhCP-A6 | A11 | XM_652272 | ATGTTTGGTTTACTCTTTGTTACTCTCATTTCATTGAGCAATGCTATTAGCTTTGATAAA |
| 11 | EhCP-A8 | B2 | XM_652354 | AATGTCCAGAAATAAGAATAATCAATGTGGTATTTGCACAGGAATTTCATTCCCAGTTGG |
| 12 | EhCP-A9 | B3 | XM_650583 | ATCAACCTGTAGCAGTATCTATTGATTCCTCACAACTAAGTTTTCAATTTTATGAAGGGG |
| 13 | EhCP-A10 | B10 | XM_646598 | GTAATAGTTGGGGTGATTGGAAATGGGGAGAAGATGGATATATGAGACTTTATAGAGGAG |
| 14 | EhCP-A11 | C1 | XM_646598 | CTTACTCGTGAAGAAAGTGTGGCTATTGCACAAGGAATTCATATAGACAAATCTGATCTT |
| 15 | EhCP-A12 | C5 | XM_648731 | CCAATTAAATCAAGTGGAACAAGATATTTTGAAGCAACAATTGATGGTATTGAAGCAGCA |
| 16 | EhCP-A13 | D2 | not annotated | CATAAATCGTACACCAACAAAGCAGAGTATCTTTATCGTCTCGCCGTCTTCTTAGACAAC |
| 17 | EhCP-B1 | B1 | XM_646489 | ACTGTTGATGGTTATGGAGAATGTGATGGACATAAATTCCTTTGGGTAAGAAATTCATGG |
| 18 | EhCP-B2 | B4 | AY156069 | GTGGATGTGGAGGAGGATTTGCTGAAGATGTTCTTGATTCTGTAGATGGAATTTATTATG |
| 19 | EhCP-B3 | B5 | XM_651655 | CTTGTGGGTCATGTTATTGTGTAAGTAATGCTCTTGCTCTTCAATTAAAATGGGCTAATC |
| 20 | EhCP-B4 | B6 | XM_643409 | AGAATAGTTATTATTGTGAGGGAGGTACTTCAGATGAACCGTTAATGTCTTCTCATTACG |
| 21 | EhCP-B5 | B7 | XM_647579 | CTGCTAAGTTATCTGCTGACAGTTTATGTGGAATAGGAAATTGTGATGGTGAGAATGTTC |
| 22 | EhCP-B6 | B8 | XM_647373 | AAACATCAAGCATTCAGCACACAACAAATCATTGATTGTTCAAACAATAATGGGTGTAGT |
| 23 | EhCP-B7 | B9 | XM_645308 | AGAGAATCACAACGATGTTATTCATGTAAAAGGTTCTATCGACTAAATCAATATTCTTGT |
| 24 | EhCP-B8 | B11 | XM_645957 | ACCATGGATAGTCGTGGTATGTGTTTAGATAATTCTTATCCTTCAATTCCAGAAGATGCT |
| 25 | EhCP-B9 | E5 | XM_647901 | CTCAAAGCATTGCGTTTAATCTAGAACTCCCCGTAGACAAATTGTTTATTCAGTTCAAAA |
| 26 | EhCP-B10 | C3 | XM_643214 | ACATTAAAATGTTCTGCTTGTAAAGCAAATACTACTCTTGATGCAAGAGGAATGTGTGTT |
| 27 | EhCP-B11 | D7 | XM_642921 | AAAGAGTATTTGACTATGATGAGAGAGCACAATGCAAAAGGAAGTTCTTATAGAATGGGA |
| 28 | EhCP-C1 | C2 | XM_649361 | ACTTTTAAACGTGGGGTATGCAAAACTAGAGAGATGGGAGATGGACTTATTGTTTATCTA |
| 29 | EhCP-C2 | C4 | XM_651540 | TCAGCATATGTTATTCCTGAAAATAGAATCACTCCAGTTAAAGGACAATTAGCTAGAGGT |
| 30 | EhCP-C3 | C6 | XM_650036 | CGTTCAACTTGTTGGTCTTTTGTTACTTCTGGTTTCTTAGAGTCTGCCTATAATTCTGAG |
| 31 | EhCP-C4 | C7 | XM_650708 | TATCCTCAATAAACATTTACCATTCTGGGGAGGGTATGTTGGAAGTCATTTTGAAATTGA* |
| 32 | EhCP-C5 | C8 | XM_649708 | TGCACCATTTATCAAAGAGAATGAAATAGTATCGACGTTTGAAAAGTCTGTAGGAAATGT |
| 33 | EhCP-C6 | C9 | XM_646461 | ACTTGTGGAGCATTTATTATTAGAGATTCTACTGATGATACTCTTGTTTATGGCAGTCAT |
| 34 | EhCP-C7 | C10 | XM_652181 | AATGGTATTAGAAAAGGATTTCTTAAAGAAAATGAATATCTTAGACTATCACCTCAAGCA |
| 35 | EhCP-C8 | C11 | XM_652181 | AGAAAGTTCATATAGAGCTTATGGTCTTCGACATAATCTTCTTAACTCAACAGAGTATGT |
| 36 | EhCP-C9 | D1 | XM_649919 | GTACCATGTCCATCATCTTTAGGAGGGGATTGTGTTGTACTTACTTTTAATCCATATTCC |
| 37 | EhCP-C10 | D3 | XM_649737 | TGCACCATTTATCAAAGAGAATGAAATAGTATCGACGTTTGAAAAGTCTGTAGGAAATGT |
| 38 | EhCP-C11 | D4 | XM_642991 | TGGGTGTCGAGATTTAGAAACGATAAATAAGTCATTTGATCCAGATTTTGCTCCTTCATT |
| 39 | EhCP-C12 | D5 | XM_645737 | TTAGTTCGTAACGAATTTCAAGGTGATTGTATTAATGACCAACTTAAAAGTGAGTGTCAA |
| 40 | EhCP-C13 | D6 | XM_651464 | GCTACCATTGGTCTTTTGGAACAATCATATAGAGATAATGACTATCACCTCAAGCATATG |
| 41 | EhCALP1 | E2 | XM_644830 | ATGTCTAAAACACCGAGAGAAAGAGCAGGAAGAGAACCTAAAATGGGAAAAGTAGGAATG |
| 42 | EhCALP2 | E3 | XM_652220 | AGCTTTAAGTGATTTAACAGGAATGCCAGTAAAACGTATATCTACCAGAGAAACAGACGT |
| 43 | EhUBHY | D10 | XM_652264 | CAGTCTTTAAAAAAGTGGTTTGAAATTAATAACGAAGAGGTGAAAGAAGCATTTTTCCCA |
| 44 | EhAUTO1 | D8 | XM_646294 | ATCACATTCACTGAACATCAACTAATTTTAGACTCATTAGCTCTTTCTCAAAGTCGAGGA |
| 45 | EhAUTO2 | D9 | XM_648706 | TTCATGGAAATTATGTCCTATTAGATGTGTTATGTGTTCAAATGTTTCAATACCAACTCA |
| 46 | EhAUTO3 | D11 | XM_646951 | ACACCTTTCAACTTTGTTTCGAATCACTTATAGAAATGGCTTTACTTACCATTTACCTCA |
| 47 | EhAUTO4 | E1 | XM_651632 | TTTCTTTGGGATACATCACAATCAATTACTTTTTCTTGACCCTCATTTTGTACGTCCATG |
| 48 | EhOTU | E4 | XM_648921 | TAAAATGGGTTCCGATTAGAAGAAAAATGGCTGCTGATAATTCCTGTTTATTTCATTGCT |
| 49 | EhSP9-1 | H2 | XM_650173 | AGGATATGATAATCAACCATTATTTAATAATGATGGAAATAAAAGTGATCAATCAGTATT |
| 50 | EhSP9-2 | H3 | XM_650130 | TGGAAGTCTTTTATATTATCTTTCAATGTCAGCACCTAAAGATGAAAGTGATAAATCAGT |
| 51 | EhSP9-3 | H4 | XM_651288 | GGTCTTACAGATGGAATTTCTTATTAATGGCATCAGAAGGGTACATTATTATTGCACCGA |
| 52 | EhSP9-5 | H5 | XM_650584 | GTTATTATATTTTCCCGACGAAAACCACTGGGTTGTTAAAGCACAAAATGGGATGTTATG |
| 53 | EhSP26-1 | H7 | XM_648050 | TATTCAAAATGTTACTCAGTTTGGATTAATTGTTGCATCTGCTGTAATATTGTGGAAAGC |
| 54 | EhSP26-2 | G3 | XM_646699 | GGTCTGGTGCTACTACTGTTGAACATGGAAATTCTACTAAATTCGTACTTCCTACTGAAT |
| 55 | EhSP28-1 | H9 | XM_651670 | CAGTTACGCAAAACTCTCACTCAACCAACAAGTAATGCAACAAATATCTCAATCATTCAT |
| 56 | EhSP28-2 | H8 | XM_643899 | TATCAATGTTAGATATGGAGGAAAGAAACCATGTGTAACCAATGTTGCATTTACAAATGG |
| 57 | EhSP28-2 | H6 | XM_651706 | TCTGATATACCAAACGTTAAAACATTGACTTATACTGTTCCATTGGATCATTTCAACGCT |
| 58 | EhSP28-3 | H10 | XM_646997 | GCATGGCAAATCTGTAGTGAATACAGTTATTTCCAACCAGTTAATGAAAGTCTTCCATTT |
| 59 | EhMP1-1 | F4 | XM_647466 | TTTGGTGATTTAGTTACAATGAAATGGTGGAATGATCTTTGGCTTAATGAAGGATTTGCT |
| 60 | EhMP3-1 | G7 | XM_644785 | TCAACTAAATTCAAAACTTCTATCCTTGAAATGGGTGGTTCTAAACCAGCAAGTGAATTA |
| 61 | EhMP3-2 | G8 | XM_644508 | AGATCAGACATTGAAGAATATCGAAAAGCATATGATGAAATTCTCCCTAAAGTAACAGAA |
| 62 | EhMP8-1 | F1 | XM_650302 | TTTTGAAGACCTAGGAACTTATTCAGTTAATTATAGTGCTGCTGAACCATTAACATGGGG |
| 63 | EhMP8-2 | F2 | XM_647540 | CTGGTGTAAGGAGGTGTACTAATGACCGTTCTGCTATTGGTATATGTGATGGAAATTCAT |
| 64 | EhMP48-1 | G4 | XM_643678 | ATGTTGTAGATCCATTTGTCTCTACAATTGAAAACTCTCACCCAAATCTTGTAGAACGAA |
| 65 | EhMP16-1 | F3 | XM_649757 | ATAAGGTTAGAGTTGAAGGTGGTGCTTATGGAAGTTGGATGTCTTATTCATATAGTGGAA |
| 66 | EhMP24-1 | G2 | XM_646447 | CAATTCATAAAAGTGTTAGACAATGGGCTCAACAATGGATTAAACCAGGAATGTCAGATC |
| 67 | EhMP24-2 | H1 | XM_651993 | AGACTCAATTATTTTCTTAGAAGGTGGTCTTGAACTTCCATTTTATGATACTGATGGTGA |
| 68 | EhMP24-3 | F8 | XM_649119 | TATTAACTGATCTTGCTGATGTTAATTGGGCTTTCAATATTAGAGCACATGATATTCCTT |
| 69 | EhMP24-4 | F6 | XM_645554 | AGAACCATTATTATTCCACGTATAAAAGATAGTTATGTAATTCAGTGGGTTGAATCTAAT |
| 70 | EhMP24-5 | F5 | XM_644888 | TGGTTCCCAATATAAAGAAGGATGTACTACTGATGTAACAAGAACTGTTCATTATGGAGA |
| 71 | EhMP24-6 | G9 | XM_648239 | TTGGAGAAGAAGCAACAGGAGATAAAGCAACTATTATTGAAGCAGGATATACTGCACTTC |
| 72 | EhMP18-1 | F7 | XM_651526 | AACTAGGAGAAGAGGTTGGAGTAAAGTTTCAAAGAACTGTTAAACGACAAGAAAAAGGAG |
| 73 | EhMP18-2 | F11 | XM_645374 | GTACAGAAAAGACTTGAGTCTGCTGGTTATGTCCGTCTTAAAGAAAATGAGGTTTGGAAC |
| 74 | EhMP20-1 | G10 | XM_651336 | AAATAATAACTTCTAGTGGTGATACATTACTTGGTGCAGACGATAAATGTGCTGTTGCTA |
| 75 | EhMP20-2 | G22 | XM_645060 | TCACCATAAATAGTCAAAGTAATGAATCAACTCATGTCACTCCTTCAACCCAATGTCAAT |
| 76 | EhMP20-3 | F9 | XM_651453 | GGAGATGATGGAACTGGAGTTGCATGTGGACTTGCATATATGGAACTTAGAGATAAATTC |
| 77 | EhMP20-4 | F10 | XM_650524 | TTTATCAGCACAAGGAACTACTCTTGGAGGAGATGATGGAACTGGAAGATAAATTCCAAC |
| 78 | EhMP22-1 | G6 | XM_647200 | ATCTAATGTCTAAGAGTGGTCAGTTTACGCCTATAGAAGATGCAACAGTACATCAAAGAT |
| 79 | EhMP49-1 | G1 | XM_649181 | CTTTAAGTCACTTGGAAGTGCAATGGAAGAGTGTAGAGCTGAAGCTGTTGGATTATTCTT |
| 80 | EhU48-1 | G5 | XM_651374 | AGAGGAGTGTTTATACCATACCTGTTGACAAACGGATGCACATCAATATTTACATTTATC |
| 81 | EhCPI-1 | E6 | XM_648163 | TGTTTTAGTATACAGAAGACCTTGGGCACCAAATGCTAATGACCGTACTTTTACTTTAAA |
| 82 | EhCPI-2 | E3 | XM_644271 | TCAACTTAGAACAAATCCATCAACAGGATATGCTTGGAATATTGAATACCCAACTGACAC |
| 83 | SP-Inhibitor1 | H11 | XM_645170 | ATTTGATGAACGTGCAGATTTTAGTAAAATGGCAAAAGGACATTTTTGTGTTTCAGAAGC |
| 84 | Actin | A1 | XM_651646 | CTACATTCCAAAACATGTGGATTACCAAGGAAGAATATGATGAATCTGGACCAGCTATTG |
| 85 | Hexokinase | E8 | XM_650873 | GGGTATTGATGTTGGTGGAACTAATCTTAGAGTCTTATTATTAGAAATCCCTGAACCTGG |
| 86 | Phosphofructokinase | E9 | XM_648631 | AAAGGTCTTGATCTTGGTAGTGATAAATCAGGAAATATTGTTCATTGGGATGCTGTTACA |
| 87 | Enolase | E10 | XM_644069 | TGAAACTGAAGATAC-TTTCATTGCTGATCTTGTTGTTGGACTTAACTGCAAACAAATCAA |
| 88 | Histon H2A | E11 | XM_647378 | AGATGTTACTATTTCTTATGGTGGAGTATTCCCTAATGTTCC-TACTGCCGTTAATTCAAA |
