# Supplementary material for: The Entamoeba histolytica genome: primary structure and expression of proteolytic enzymes
Source: BMC Genomics. 2007 Jun 14;8:170. doi: 10.1186/1471-2164-8-170 (PMC1913524; doi:10.1186/1471-2164-8-170)
Supplement: Additional File 2 — List of oligonucleotides used for RT-PCR. The table shows a list of all oligonucleotides used for RT-PCR. [file 1471-2164-8-170-S2.doc]

Additional file 2: List of oligonucleotides used for RT-PCR

| **No** | **Protein**  **name** | **Accession No.** | **Oligonucleotide sequence (5’)** | **Oligonucleotide sequence (3’)** |
| --- | --- | --- | --- | --- |
| 1 | AsP22-1 | XM_648987 | AACTCATGTTGACGGACCAA | TGCAATATCACCAAGTCCAAG |
| 2 | AsP22-2 | XM_647728 | TCCAGGAATGTTCATTACAC | AATCATTGCTGGAACTATGC |
| 3 | AsP22-3 | XM_652471 | TTATTGCTATTGGAGTTGCAG | TTGCAGCTTCAACCATTACT |
| 4 | AsP22-4 | XM_648604 | TTGGTGTTCTTGGAGTCATCTC | AAGCCATGAAGACGCTGATA |
| 5 | EhCP-A1 | XM_645064 | TGCATCATCTGTTCAATTCC | CAACACCATATCCAACAGCA |
| 6 | EhCP-A2 | XM_645550 | ATCCAAGCACCAGAATCAGT | TTCCTTCAAGAGCTGCAAGT |
| 7 | EhCP-A3 | XM_648162 | AATGGATGTGAAGGAGGACA | GCAAGTACCAGCAACAGCTT |
| 8 | EhCP-A4 | XM_651510 | CAGAAGGACCAGTTGCTGTT | ATATCCTACAGCGGCAACAC |
| 9 | EhCP-A5 | XM_645845 | CCAGAATCTGTTGATTGGAGA | GCAACCAACAATCTTCCTTC |
| 10 | EhCP-A6 | XM_652272 | TTGCTATTGATGCAGGTCAA | AGATCCATATCCAACAGCACA |
| 11 | EhCP-A7 | XM_643904 | ATTGCAGCACTTGAAGGAAG | AATCCTCCTCCACATCCATT |
| 12 | EhCP-A8 | XM_652354 | TTGCAGCAATTGAATCAAGA | GCCTGAAGAGTTCCTCCATT |
| 13 | EhCP-B9 | XM_647901 | GAATGGCTATTCCTGTCTCTTC | TAATGGCGGTACTTCATTCC |
| 14 | EhSP9-2 | XM_650130 | GACCAGAATCACCATGGACT | TGCATCTCCATAACTTCCTGA |
| 15 | EhSP28-1 | XM_651670 | GAAGGACCTGCCTCTCCTAA | GATGGAGTTGAAGCACCATAA |
| 16 | EhSP28-2 | XM_643899 | CCAGTTATTGTTCTTGGTGGA | TTCTACTGGAGCAGATGATGC |
| 17 | EhSP28-4 | XM_646997 | TGTGCACCATCAAGTTATGC | CCAACTTCTTGTTGATGCAAG |
| 18 | EhMP8-1 | XM_650302 | GTGCTGCTGAACCATTAACA | TCCAATGGCTCCTCTATCAG |
| 19 | EhMP8-2 | XM_647540 | AATGACCGTTCTGCTATTGG | CAGCAATCTCATCACTTCCA |
| 20 | EhMP48-1 | XM_643678 | GGTAATCATCAATTAGCAAG | TTGCTTGTATTCGTTCTACA |
| 21 | EhMP20-3 | XM_651453 | GTTGATGATGCATGTGGATG | TTCAAGACCAGCATGAATTG |
| 22 | EhMP49-1 | XM_649181 | TGGTGCAGTTGAAGTTGTTG | CTGTGTCAGCAGTGGCTCTA |
| 23 | Actin | XM_651646 | AAGCTGCATCAAGCAGTGAA | GGAATGATGGTTGGAAGAGG |
